# Efficacy and safety of Zhen Wu Decoction against chronic heart failure: A protocol of randomized, double-blinded, and controlled trial

**DOI:** 10.1097/MD.0000000000027260

**Published:** 2021-09-24

**Authors:** Jian Liang, Xianming Tao, Dabi Hu, Yi Cao

**Affiliations:** The People's Hospital of Dazu, Chongqing Dazu, Chongqing, China.

**Keywords:** Chinese medicine, chronic heart failure, LVEF, protocol, Zhen Wu Decoction

## Abstract

**Introduction:**

Chronic heart failure (CHF) is the end stage of several cardiac diseases. 50% of patients with severe CHF would survive less than 1 year, which has seriously affected patients’ survival and quality of life. The current modern therapy could improve survival and quality of life. However, a larger number of patients still suffer from repeated hospitalization, unsatisfactory efficacy, and many side effects. In China, Zhen Wu Decoction (ZWD), a classic prescription recorded in *Treatise on Febrile Diseases*, was widely used for CHF. In this study, we try to conduct a prospective, double-blinded, randomized, controlled study to evaluate the efficacy and safety of ZWD in the treatment of CHF patients in China.

**Methods:**

Patients will be randomly divided into treatment group and control group in 1:1 ratio. Guideline directed medical therapies and ZWD will be provided for patients in treatment group, while guideline directed medical therapies and ZWD-granules simulations for control group. Left ventricular ejection fraction, left ventricular end diastolic diameter, left ventricular end systolic diameter, b-type natriuretic peptide, NT-proBNP, peak VO2, VO2 maximum, exercise time, and walking distance will be recorded. The data will be analyzed by SPSS 22.0.

**Conclusions:**

The results will evaluate the efficacy and safety of ZWD in the treatment of CHF patients.

**Trial registration:**

OSF registration number: DOI 10.17605/OSF.IO/G3QNU.

## 1 Introduction

Chronic heart failure (CHF) is a group of manifestations caused by either cardiac structure change or heart dysfunction resulting in impaired ventricular filling and/or ejection ability.^[1–3]^ CHF is the end stage of several cardiac diseases, include coronary artery disease, high blood pressure, valvular heart disease, and atrial fibrillation. The prevalence of CHF is increasing and it is predicted that there will be 8 million patients by the year 2030 in the USA.^[4]^ CHF patients commonly suffer from fatigue, shortness of breath, excessive tiredness, and leg swelling that limit activities in daily life. It is a potentially fatal condition, and about 35% might die in the first year after diagnosis.^[5]^ 50% of patients with severe CHF would survive less than 1 year, which has seriously affected patients’ survival and quality of life.

CHF is the leading reason of older adults for hospital admission.^[4]^ At present, treatment against CHF still focuses on the improvement of the symptoms and prevention of the progression. Common standard therapy includes angiotensin converting enzyme inhibitors (ACEI) or angiotensin receptor blocker (ARB), β-receptor blockers, aldosterone receptor antagonists, inotropic drugs, and diuretics.^[3]^ The current therapy could improve survival and quality of life. However, a larger number of patients still suffer from repeated hospitalization, unsatisfactory efficacy, and many side effects. Though cardiac resynchronization therapy has been rapidly developed, it is expensive and difficult for most patients to accept, particularly in low- and middle-income countries.

Traditional Chinese medicine could significantly improve the clinical symptoms, delay the development of the disease, and improve the long-term prognosis of CHF patients based on the common modern therapy.^[6–9]^ In China, Zhen Wu Decoction (ZWD), a classic prescription recorded in *Treatise on Febrile Diseases*, was widely used for CHF.^[10]^ However, it is still lack of high-quality and well-designed randomized, controlled trial to evaluate its efficacy and safety against CHF. Therefore, in this study, we try to conduct a prospective, double-blinded, randomized, controlled study to evaluate the efficacy and safety of ZWD in the treatment of CHF patients in China.

## 2 Methods

### 2.1 Patients selection

The double-blinded, randomized, controlled trial will be carried out in The People's Hospital of Dazu, and it has been approved by the Health Research Ethics Board of The People's Hospital of Dazu. The protocol conforms to SPIRIT 2013 Statement,^[11]^ and the results will be reported according to the CONSORT Statement extension for trials.^[12]^ The study was registered in open science framework (registration number: DOI 10.17605/OSF.IO/G3QNU). All patients will provide a signed written informed consent at enrollment.

Patients aged over 18 years with CHF in stable condition, New York Heart Association functional class II or III, left ventricular ejection fraction of <40% will be recruited. Patients will be excluded with the following criteria:

(1) new onset atrial fibrillation or atrial flutter;
(2) complex ventricular arrhythmia at rest or presenting with exertion;
(3) serious liver and kidney function damage or malignant tumor;
(4) cognitive or neurological problems;
(5) respiratory infection in the previous 30 days.

### 2.2 Randomization and blinding

**Figure 1:**
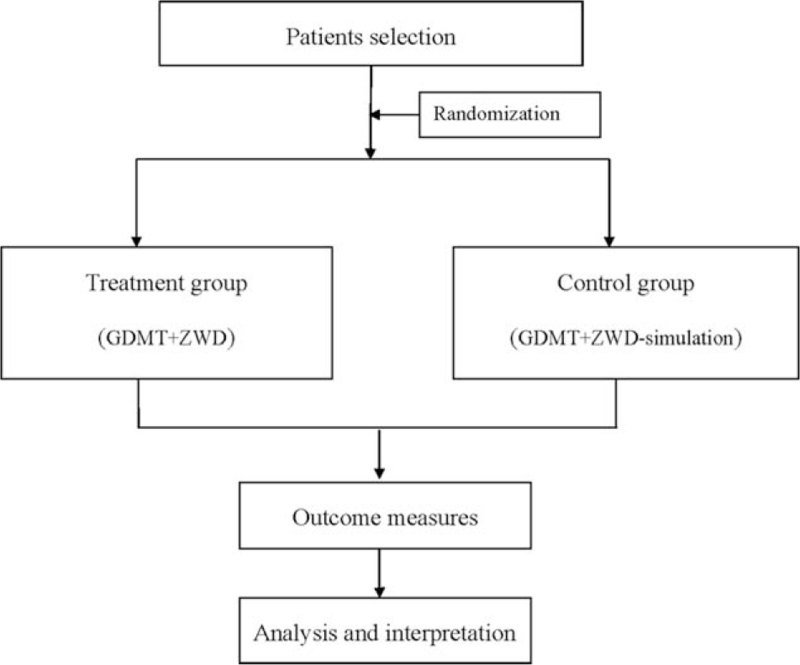
Flow diagram of the trial.

Patients will be randomly divided into treatment group and control group with a 1:1 ratio (Fig. 1). The randomization sequence will be generated by an independent statistician. The doctors, patients, and outcome assessors will be blinded to the allocation.

### 2.3 Interventions

According to *Guidelines for Diagnosis and Treatment of Heart Failure in China 2018*,^[13]^ all enrolled patients will take standard guideline directed medical therapies, with a mix of ACEI or ARB, beta blockers, sacubitril/valsartan, and mineralocorticoid receptor antagonist.

In the treatment group, patients will take Chinese herbal formula ZWD, composed of *Radix Aconiti Carmichaeli* (Fuzi) 10 g, *Radix Paeoniae Alba* (Baishao) 10 g, *Rhizoma Atractylodis Macrocephalae* (Baizhu) 10 g, *Poria cocos* (Fuling) 10 g, *Rhizoma Zingiberis Recens* (Shengjiang) 10 g. The herbs will be provided as concentrate-granules and quality controlled by Beijing Tcmages Pharmaceutical CO., Ltd. The patients will be taught to dissolve the granules in 50 mL and orally take it, twice a day, for 14 days.

While in the control group, ZWD-granules simulations will be taken twice a day, for 14 days. The simulations, composed of amylum, share the same appearance, taste, dosage, and administration methods with the formula in the treatment group.

### 2.4 Outcome measures

The primary outcome is the change of left ventricular ejection fraction during the treatment. The secondary outcomes are left ventricular end diastolic diameter, left ventricular end systolic diameter, b-type natriuretic peptide, NT-proBNP, peak VO2, VO2 maximum, exercise time, and walking distance. All adverse events will be also recorded.

### 2.5 Sample size

In our preliminary trial, the change of left ventricular ejection fraction in the treatment group is 19%, and 10% in the control group. Taking α = 0.05, β = 0.2, 27 patients will be needed in each group. Considering a loss to follow-up of 10%, the estimated total sample size will be 60.

### 2.6 Discontinue and data monitoring

The supervisor will decide for discontinuing. Case report forms (CRFs) will be used in data collection to record demographics, assessment, reasons of patient drop out. All case report forms will be preserved for at least 5 years, and the access to the original data are available from the corresponding author on reasonable requests.

### 2.7 Statistical methods

The data will be analyzed by SPSS 22.0 (IBM, Chicago), with presented as mean ± standard deviation for measurement data, and percentages for count data respectively. Independent-samples *T* test, Mann–Whitney *U* test, *χ*^2^ test, or Fisher's exact test will be applied accordingly. *P* values less than .05 will be considered statistically significant.

## 3 Discussion

CHF is the final stage in the development of various heart diseases. Its morbidity and rehospitalization rate are high, which seriously affects patients’ health and quality of life.^[14,15]^ The standard therapy of beta blockers, ACEI, or ARB could improve the survival rate of patients. However, long-term western medicine treatments may have many adverse reactions. With the advantages of multi-target, multi-component, multi-pathway, traditional Chinese medicine has been widely applied in the treatment of CHF.^[10]^ It could not only improve the clinical symptoms, but also has fewer side effects. In China, ZWD is a commonly used herbal formula for heart failure caused by various heart diseases such as coronary heart disease, rheumatic heart disease, and dilated cardiomyopathy. However, it is still lack of high-quality and well-designed randomized controlled trials to evaluate the efficacy and safety of ZWD against CHF. In this study, we try to conduct a prospective, double-blinded, randomized, controlled study to evaluate the efficacy and safety of ZWD in the treatment of CHF patients in China.

## Author contributions

**Data collection:** Jian Liang, Xianming Tao.

**Funding support:** Yi Cao.

**Investigation:** Xianming Tao.

**Literature retrieval:** Jian Liang, Dabi Hu.

**Software operating:** Yi Cao, Dabi Hu.

**Supervision:** Dabi Hu.

**Writing – original draft:** Jian Liang, Xianming Tao.

**Writing – review & editing:** Jian Liang, Yi Cao.
